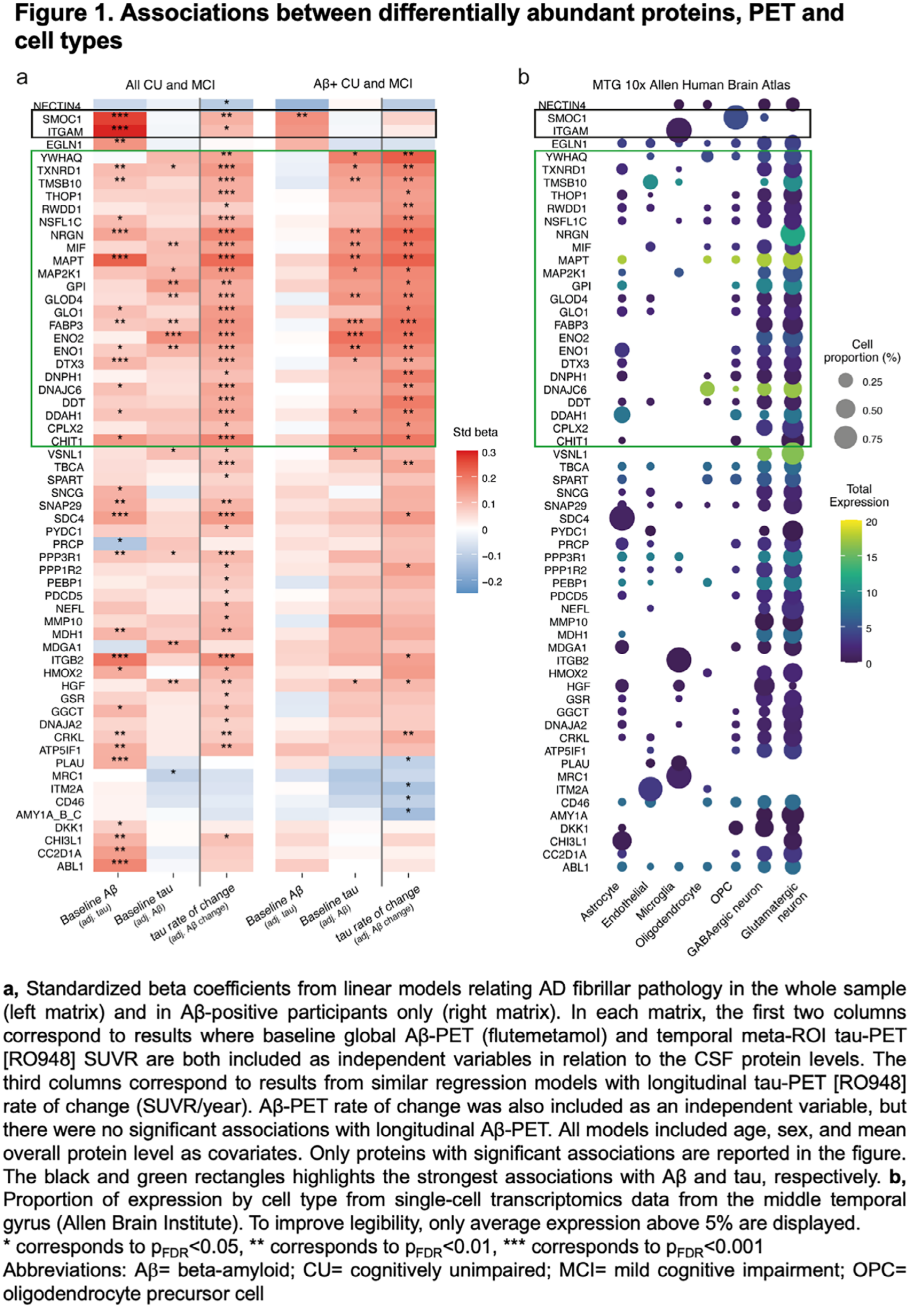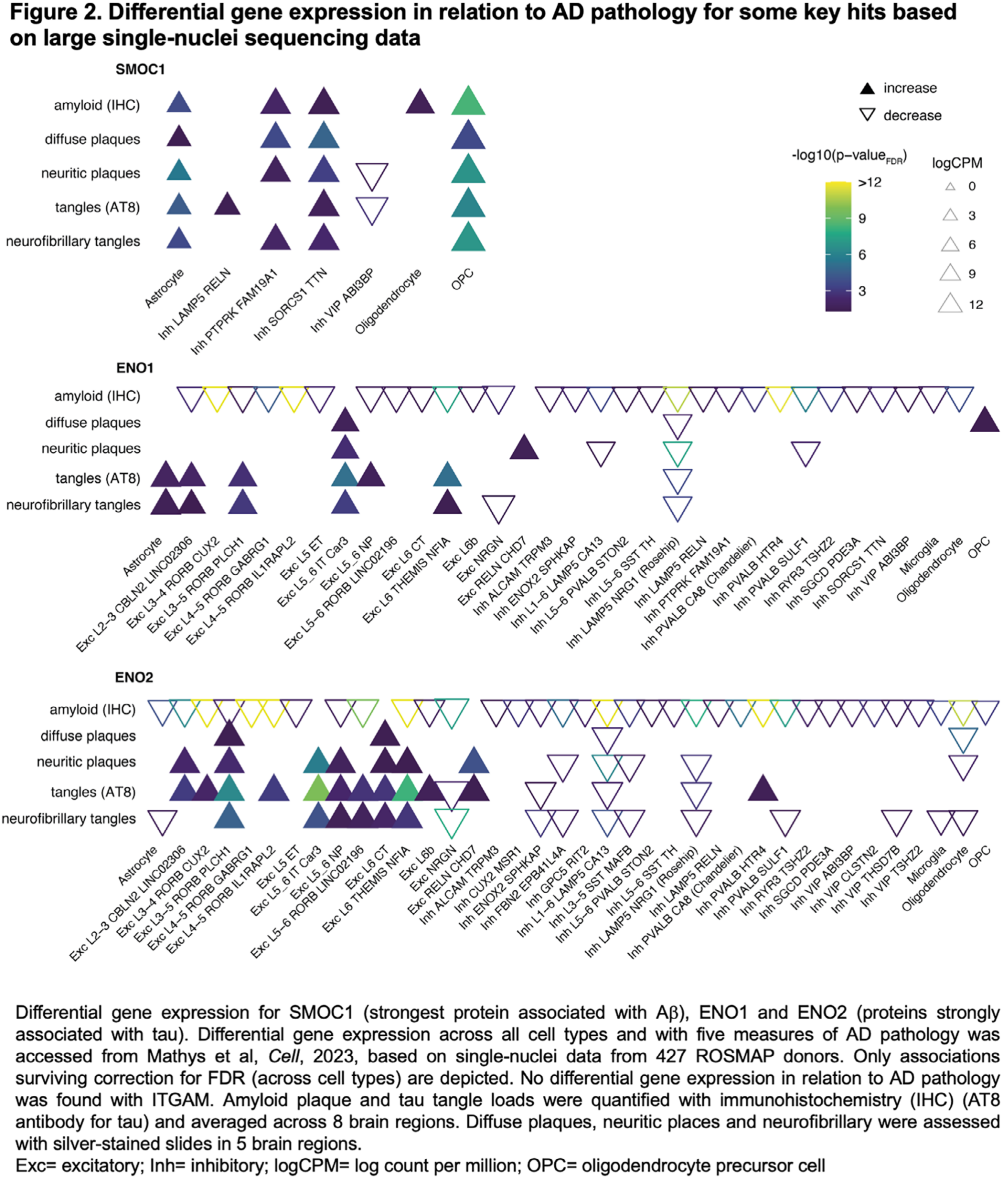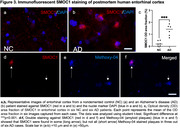# Proteomics analysis characterizing new markers related to Aβ plaques and tau tangles pathologies

**DOI:** 10.1002/alz.091288

**Published:** 2025-01-09

**Authors:** Alexa Pichet Binette, Malin Wennström, Olof Strandberg, Hansruedi Mathys, Li‐Huei Tsai, Niklas Mattsson‐Carlgren, Erik Stomrud, Shorena Janelidze, Jacob W. Vogel, Oskar Hansson

**Affiliations:** ^1^ Clinical Memory Research Unit, Lund University, Lund Sweden; ^2^ Lund University, Malmö Sweden; ^3^ Clinical Memory Research Unit, Department of Clinical Sciences Malmö, Faculty of Medicine, Lund University, Lund Sweden; ^4^ University of Pittsburgh, Pittsburgh, PA USA; ^5^ Massachusetts Institute of Technology, Cambridge, MA USA; ^6^ Picower Institute, MIT, Cambridge, MA USA; ^7^ Wallenberg Center for Molecular Medicine, Lund University, Lund Sweden; ^8^ Memory Clinic, Skåne University Hospital, Malmö Sweden; ^9^ Department of Clinical Sciences Malmö, SciLifeLab, Lund University, Lund Sweden

## Abstract

**Background:**

Several studies have recently emerged describing relationships between cerebrospinal fluid (CSF) proteins and beta‐amyloid (Aβ) and tau pathology. While these studies have primarily characterized Alzheimer’s disease (AD) proteinopathies using CSF markers, positron emission tomography (PET) more accurately captures these pathologies, especially fibrillar tau pathology. Our objective was to identify the main proteins strongly associated with AD pathology measured by PET, and to further investigate their cellular role using postmortem transcriptomics and immunohistochemistry.

**Method:**

We included 604 BioFINDER‐2 participants with CSF measurements of proteins (Olink Explore3072) at baseline, and Aβ‐PET and tau‐PET at baseline and longitudinally. We fitted linear regressions between protein levels as outcome and (1) global Aβ‐PET and temporal meta‐ROI tau‐PET SUVR or (2) their rate of change as independent variables. Enrichment analyses were performed to characterize biological processes related to the set of significant proteins. Single‐nuclei sequencing datasets were used to investigate cell‐type specificity and differential gene expression of the main proteins in relation to AD pathology.

**Result:**

Analyses were restricted to 127 differentially abundant proteins that we previously identified as related to AD (manuscript in revision). ITGAM and SMOC1 showed the strongest associations with Aβ‐PET load, with SMOC1 being the only protein showing an association in Aβ‐positive participants only (Figure 1a). SMOC1 was mainly expressed in oligodendrocyte precursor cells (OPCs), and ITGAM in microglia (Figure 1b). In a large single‐nuclei human dataset, SMOC1 gene expression was increased with greater AD pathology primarily in OPCs (Figure 2), and immunohistochemistry revealed accumulation in Aβ plaques (Figure 3). Higher baseline tau‐PET and greater longitudinal change were associated with a set of 15 proteins, particularly in Aβ‐positive participants (Figure 1a). Such tau‐related proteins were mainly expressed in neurons (Figure 1b) and enriched for two terms associated with glycolysis, from which the key contributing genes were ENO1, ENO2 and GPI. In single‐nuclei sequencing data, ENO1 and ENO2 gene expression were increased with greater tau tangle pathology primarily in neurons (Figure 2).

**Conclusion:**

Through comprehensive proteomics analysis of CSF proteins recently found to be associated with AD, we identified differential associations with Aβ plaques and tau tangles, and further corroborated such findings in postmortem data.